# Supplementary material for: Can perceptuo-motor skills outcomes predict future competition participation/drop-out and competition performance in youth table tennis players? A 9-year follow-up study
Source: PLoS One. 2023 Feb 10;18(2):e0281731. doi: 10.1371/journal.pone.0281731 (PMC9916551; doi:10.1371/journal.pone.0281731)
Supplement: Attachment — Submitted filename: 20221030_Response to reviewers_PONE_IF.docx [file pone.0281731.s001.docx]

**Point-by-point response**

**Journal Requirements:**

When submitting your revision, we need you to address these additional requirements.

1. Please ensure that your manuscript meets PLOS ONE's style requirements, including those for file naming. The PLOS ONE style templates can be found at

https://journals.plos.org/plosone/s/file?id=wjVg/PLOSOne_formatting_sample_main_body.pdf and

https://journals.plos.org/plosone/s/file?id=ba62/PLOSOne_formatting_sample_title_authors_affiliations.pdf.

2. We note that you have stated that you will provide repository information for your data at acceptance. Should your manuscript be accepted for publication, we will hold it until you provide the relevant accession numbers or DOIs necessary to access your data. If you wish to make changes to your Data Availability statement, please describe these changes in your cover letter and we will update your Data Availability statement to reflect the information you provide.

3. Your ethics statement should only appear in the Methods section of your manuscript. If your ethics statement is written in any section besides the Methods, please delete it from any other section.

**Response**

We have addressed all points to the best of our knowledge, and have revised the manuscript accordingly. Since these revisions were not substantive changes, they are not indicated as tracked changes within the manuscript to maintain readability.

We need to change our data availability statement. The data of this study cannot be made publicly available for ethical and legal reasons; the public availability would compromise confidentiality and/or participant privacy. The data contains potentially identifying athlete information. This restriction is imposed by the Netherlands Table Tennis Association. Data will only be available on request and can be sent to the Netherlands Table Tennis Association using the email address jong@tafeltennis.nl.

The ethics statement is now only stated within the methods section.

**Reviewers' comments:**

**Reviewer #1: Can perceptuo-motor skills assessment outcomes predict future competition participation/drop-out and performance level in youth table tennis players? An explorative 9-year follow-up study**

First of all, the reviewer would like to thank the authors for their work and efforts in trying to improve sports science knowledge. The authors are commended on their efforts thus far. The article is an interesting approach to assessment outcomes predict future competition participation/drop-out and performance level in youth table tennis players. The study is well designed and well-written, with a great original article evaluating the usefulness of the topic.

**Response**

Thank you for your positive response regarding our paper and the feedback that helps to improve its content and readability.

Abstract

Line 51: eye-hand or eye hand please check and fix the throughout the manuscript

**Response**

Thank you for you careful reading. We have changed eye hand to eye-hand throughout the manuscript.

Introduction

This section is well designed and well-written. However, here is the alternative sentence for the research questions.

Line 117-122: The purposes of this study were (a) can the outcomes of the perceptuo-motor skills assessment predict competition participation/drop-out in young table tennis players, (b) can the outcomes of the perceptuo-motor skills assessment predict future performance level in young table tennis players (c) relationships between psychophysiological responses and locomotor demands.

**Response**

Thank you for your suggestion. However, we are a little bit puzzled by the alternative, especially regarding the inclusion of question (c) relationships between psychophysiological responses and locomotor demands. We believe that this was beyond the scope of our article. Nevertheless, we tried to simplify the research question, please see line 107-112.

Methods section

Line 156: no need this info https://doi.org/10.1371/journal.pone.0149037.s001. Please extract it.

What about maturation. If you do not measure it, please add limitations.

**Response**

We have deleted the link and added the maturation as a topic to the limitation paragraph (line 317-319) as proposed.

Results section: Results and tables are well shown

Discussion section: Overall the discussion is well-written and incorporates relevant literature.

References: References are well selected by the authors

Figures and Tables: This section is well designed and well-shown.

**Response**

Thank you for these compliments and again for your feedback.

**Reviewer #2: Reviewer Comments:**

**Can perceptuo-motor skills assessment outcomes predict future competition**

**participation/drop-out and performance level in youth table tennis players? An explorative 9-year follow-up study: PONE-D-22-23471**

GENERAL COMMENTS:

Thank you for your contribution to PLoS One. Overall, the paper which presents a study analysing the (prognostic) validity of motor performance in young table tennis players falls within the scope of the journal and should be of interest to the readership. Strengths of the study mainly relate to the investigation of important talent predictors at a young age (7-11) years for long-term success while utilizing a 9-year prognostic period in a sport where talent research is still scarce. However, in its current form the manuscript also presents a range of concerns in regard to its content (rationale and theoretical background of assessed predictors, definition of criterion variables, discussion of results) and presentation (particularly description and clarity of utilized methodology).

**Response**

Thank you for your positive response and critical feedback that helps us to improve our paper.

With regards to the content of the paper, some aspects require improvements.

1) First, I would ask you to elaborate on the explanation of the assessed constructs in the introduction. It should be more highlighted that the constructs you are assessing cover only some small pieces of the multifaceted talent characteristics needed for an excellent young table tennis players. Maybe you could just use a theoretical model of talent development (like the one of Gagné) or a heuristic model of talent predictors (although I am currently only aware of a soccer-specific one by Williams and Reilly, 2000) in order to show the complexity of talent in the sport of table tennis and then present the area you want to focus on. Why exactly those motor skills were chosen? Please provide rationale in the introduction.

**Response**

Thank you for your suggestions. Indeed, our starting point is that talent development is a multidimensional process in which many factors play a role. We now emphasize on that in the introduction in line 86-90 by including the conceptualization of talent as proposed by prof. Baker and colleagues. Additionally, psychomotor skills are important elements of performance in the sports domain and especially in technique-based sports like table tennis. We have added a more detailed rational about the assessed constructs (line 93-98).

Added references:

- Baker J, Wattie N, Schorer J. A proposed conceptualization of talent in sport: The first step in a long and winding road. Psychology of Sport and Exercise. 2019;43:27-33.
- Faber IR, Bustin PM, Oosterveld FG, Elferink-Gemser MT, Nijhuis-Van der Sanden MW. Assessing personal talent determinants in young racquet sport players: a systematic review. Journal of sports sciences. 2016;34(5):395-410.
- Faber IR, Nijhuis-Van Der Sanden MW, Elferink-Gemser MT, Oosterveld FG. The Dutch motor skills assessment as tool for talent development in table tennis: a reproducibility and validity study. Journal of sports sciences. 2015;33(11):1149-58.

2) Much more important are my concerns with regard the separation of the two research questions (RQ) and the utilized criterion variables for those. to purpose 3 (validity of physical tests for in-game soccer performance). It is not entirely clear to me, why exactly the two RQs were differentiated and chosen. What is RQ1 for, what is the aim of RQ2 – both are evaluating the long-term prognostic validity, by focussing potentially different things – participation and performance level. If this is the reason, this should be explained in more detail. Potentially the lacking clarity is also a result of some inconsistencies in the use of the chosen criterion: while for RQ2 future performance level is used for instance in the title, sometimes also competition performance or competition performance level are used. I would recommend being consistent here. By the way: Please think about labelling your two RQs as RQ1 and RQ2 and please use that structure (including subheadings) also for the methods and results part. This could facilitate readability.

**Response**

Indeed, what you describe is exactly what we aimed for. We wanted to evaluate the prognostic value for both competition participation and competition performance. We provided more detailed information on this at the end of the introduction section (line 98-101). We also now use RQ1 and RQ2 for the research questions as you suggested (line 107-112) and the structure of the results section. Also, we checked the consistency of terms regarding competition performance throughout the paper.

3) Further, there are some severe issues with the description and the clarity of the utilized statistical analyses. Although I generally agree with the choice of them, a final decision of the adequate use is not possible due to some missing information on the analyses and results you present. Please exactly describe and state the procedure you were following in each of your research question regarding the analyses: why and how did you exactly perform Cox regression analysis in the univariate, how in the multivariate approach. Why is the use of Cox regression (what I appreciate) useful and appropriate for your setting. Just to give you an example: It ist not clear by the means, what the survival curve in Figure 1 exactly display/how it was computed. Is it a result of the multivariate Cox regression or is it “just” the Kaplan-Meier Curve with survival rates and hazard ratios computed based on “drop-outs” in specific time intervals. Please elaborate, otherwise it is not possible to follow your results.

**Response**

As the reviewer correctly noticed, Figure 1 is a Kaplan-Meier curve which shows the survival function (i.e., probability to remain in competition) against time (i.e., the age of a player). This information is now added to the methods section (line 202-203) and results section (line 233-234).The x-axis title is changed for clarity into age (years) and for the y-axis to survival probability. The Cox regression analyses are first conducted for each item separately (univariable), to highlight the individual interest of each of the measured variables. After that also multivariable analyses were conducted.

4) The same applies for RQ2 [performance level]: which multilevel analyses did you use? Multiple Regression? What are your levels (are there some?) and why this is intended. If so, where are the random effect that were estimated /why were they not?

**Response**

Additional information is provided in the statistical paragraph of the methods section (line 206-215) to better explain the analyses that were conducted.

5) Furthermore, with regard to the utilized criterion variables, please clarify how exactly performance level was measured. There is some information given about a score, but this is not detailed enough. Please elaborate and please also refer to the former comment regarding consistency in labelling/defining your main outcomes/criterions in the former comment.

**Response**

As proposed, we added information about the competition rating score in line 185-194. Hopefully, this provides enough information. Please let us know, whether specific information is still missing.

6) Moreover, I really struggle with your approach to aggregate performance outcomes of boys and girls. I know, that this was followed due to the rather low sample size and I appreciate reading about this short-coming in the discussion (and considering gender at least as a covariate). However, I think you should enlarge this part of the discussion by giving some information on potential similarities or dissimilarities of talent pathways/ differences in performance/ as to whether the utilized performance scores are comparable among gender. Apart from that, I would really like to see the results of the utilized covariates e.g., gender also in your tables. There are only some hints on covariates without providing the results for them.

**Response**

Although we indeed agree that an analysis including sex would be the better solution, this is considered inappropriate while including a small dataset. Therefore, we only corrected for sex by including sex as a covariate. It is quite common not to report the coefficients and p-values of the covariates in a regression model. The coefficients of the covariates are not part of the research question and therefore, they don't provide relevant information. That's the reason why the coefficient for age and sex were not reported. Anyway, if you still want to see them, of course, we can provide, but again, we don't believe that this information is very relevant. In addition to this, we emphasize in the discussion part on a future approach in which sex is taken into account (line 291-293; 312-319; 326-328).

SPECIFIC COMMENTS

Title

• “assessment outcomes” sounds a bit confusing- only assessments or only outcomes or just diagnostics?

• Why explorative? Please add a rational in the text. Is this important for the title?

Abstract

• L.44: decision-making: think about replacing by making decisions regarding selection/promotion (or similar). Decision-making is connoted with making in-game decisions and therefore could be misleading here.

• L.46: competition performance vs. future performance level? See general comment

• L.54: add regression?

**Response**

We have made changed to the manuscript as proposed:

- Assessment is deleted.

- Explorative Is deleted; this was mainly due to the small sample, but seems unnecessary.

- Decision making is changed into making decisions or similar wording throughout the paper to avoid misunderstandings.

- Competition performance is used throughout the document.

- Regression is added to the description.

Introduction

• L.66: please define excellent adult performance

• L.72: provide reference

• L.75: I agree that longitudinal approaches are needed. But, please be aware that your approach is prospective, but in my point of view not longitudinal – in the sense that you assessed predictors only once. Please precise.

• L.78 and L.90: what do you mean by fair estimate /evaluation of potential. This should be explained (also it might be implicitly clear)

• L.81: maybe add that talent development programs are established approaches of talent promotion in a variety of sports with some examples, the focus on table tennis)

• L117: explain, why the study is explorative

**Response**

The comments have been addressed as following:

- Excellent adult performance is defined as international level according to the classification levels of Swann (2014) (line 46) with reference to: Swann C, Moran A, Piggott D. Defining elite athletes: Issues in the study of expert performance in sport psychology. Psychology of sport and exercise. 2015;16:3-14.

- The following reference has been added (line 53): Faber IR, Sloot L, Hoogeveen L, Elferink-Gemser MT, Schorer J. Western Approaches for the identification and development of talent in schools and sports contexts from 2009 to 2019-a literature review. High Ability Studies. 2021:1-34.

- This text has been changed into: prospective longitudinal approaches including longitudinal tracking in line with the scoping review of Baker et al. (line 54 and line 73-75).

- More details have been added to clarify the estimate of potential: A fair estimate of an athlete’s potential to reach the international level and the chances to stand on international podia…. (line 59-61)

- The suggested sentence is added (line 63-64) together with the following reference: De Bosscher V, De Knop P, Van Bottenburg M, Shibli S. A conceptual framework for analysing sports policy factors leading to international sporting success. European sport management quarterly. 2006;6(2):185-215.

- Explorative Is deleted; this was mainly due to the small sample, but seems unnecessary.

Methods

• l.144 provide distribution regarding gender and age

• Is there a rule, in which ages the players are nominated for the squads? If I am right, players were between 7 and 11 years old, which seems to be a rather large range for initial talent identification events (if the events were of such type?)

• L.157pp: please double check the presentation of the statistical values. Should read e.g., ICC = 0.91. Please also check formatting (e.g., n should be in italics, see for instance l.144)

• L166-168: sentence seems not to be complete.

• L.170pp: Please explain which “levels” were exactly used (or whether level is the right term for the score that was used). See also general comment.

• L.187: was there also significance testing for normality?

**Response**

The comments have been addressed as following:

- The distribution of gender and age is added to the results section (line 219).

- In table tennis it is common practice that players are selected for more intensive training programs already at an early age, e.g., from 10-12 years or even younger. The context presented within the paper is representative for a common situation in the Netherlands. The first time that players are selected for international competitions (i.e., European Youth Championships) is for the under-15 category.

- Statistical values were adapted as proposed (line 162-168).

- Sentence has been reformulated (line 173-175).

- We deleted the word level and changed this into competition performance. The competition rating score is used to reflect the competition performance of a player. More detailed information of the score is added (line 185-194).

- We didn’t perform a statistical test for normality. The normality tests are not very valid for small sample sizes. Therefore, we evaluated normality by comparing mean and median and by visual inspection.

Results:

• L.215: lower values are better performances in speed measures, right? Please note on that, as this is important for the interpretation of the HRs.

• By the way: although I like the idea of referring to the diagnostics elsewhere, it would be helpful to at least provide the units /general shape of the utilized tests) in order to better understand interpretations in this part.

• “best fitting model”: please provide measures for that (and describe the process how it was found in more detail)

• L.226pp: Please state what was used as independent and dependent variables in the regressions (already in the methods section) and add this information also in Table 2.

**Response**

The comments have been addressed as following:

- This information is added as footnote in Table 1.

- More detailed information about the tests is added to the methods section (line 144-160)

- After considering this formulation, we think it's better to approach this as the final multivariable model instead of the 'best fitting' model. We used a backwards procedure to come to this final model and did not make a comparison. We changed this accordingly.

- The information about independent and dependent variables is now added to the methods section within the statistical analysis paragraph (line 203-208).

Discussion

L.250: decimal point instead of comma

L.261: commas before and after “therefore”?

l.290: see general comment on combined approach regarding gender

L.195pp: In line with my comment regarding the introduction of the utilized and investigated measure, I would recommend to extend this paragraph by giving more information why the study “only” focused on a small part of the complex construct of talent and to highlight that this small piece is more a support for coaches and not a replacement (?).

**Response**

The comments have been addressed as following:

- This is changed as proposed (line 276).

- This is changed as proposed (line 286).

- Although we indeed agree that an analysis including sex would be the better solution, this is considered inappropriate within this small dataset. Therefore, we only corrected for sex by including sex as a covariate and included this in the limitation paragraph of the discussion section.

- This information is added to the concluding paragraph (line 320-326).

Figure 1:

• See general comment. I would recommend deleting ages 0-7 years (not included in the study, and players were potentially not in the system yet. Please also provide Confidence intervals for the survivals (in order to get insight in potential problems due to lower sample sizes for later drop outs). Please add also a label for the columns with n=21 and n=18 (presumably the 2011 and 2012 assessments separately?)

**Response**

The comments have been addressed as following:

- Ages from 0-7 are deleted as proposed in Fig1.

- The Kaplan Meier is a descriptive plot to display observed values. For this reason, confidence intervals cannot be calculated. Because they are observed values, you have no CI.

- Column labels have been added to Table 1.

Once again, thank you for your feedback that helped us to improve our article.
