# Supplementary material for: Can perceptuo-motor skills outcomes predict future competition participation/drop-out and competition performance in youth table tennis players? A 9-year follow-up study
Source: PLoS One. 2023 Feb 10;18(2):e0281731. doi: 10.1371/journal.pone.0281731 (PMC9916551; doi:10.1371/journal.pone.0281731)
Supplement: Attachment — Submitted filename: 20230117_Response to reviewers.docx [file pone.0281731.s002.docx]

**Point-by-point response**

**Journal Requirements:**

Please review your reference list to ensure that it is complete and correct. If you have cited papers that have been retracted, please include the rationale for doing so in the manuscript text, or remove these references and replace them with relevant current references. Any changes to the reference list should be mentioned in the rebuttal letter that accompanies your revised manuscript. If you need to cite a retracted article, indicate the article’s retracted status in the References list and also include a citation and full reference for the retraction notice.

**Response**

We checked the references. No updates were necessary for this revision.

**Additional Editor Comments:**

Dear authors,

The authors improved their work when compared with the previous version. Reviewer 1 already recommended acception of the work while reviewer 2 suggested minor revision. This is also my opinion because there are still some possibilities to developt the content of the manuscript. Please check all comments made by reviewer 2 and address all of them. I believe that if all is satisfied, the paper will be accepted.

**Response**

Thank you for your positive response regarding our paper.

**Reviewers' comments:**

**Reviewer #1:**

First of all, the reviewer would like to thank the authors for their work and efforts in trying to improve sports science knowledge.

**Response**

Thank you for your positive response regarding our paper and accepting it for publication.

**Reviewer #2:**

Thank you for submitting your revised manuscript to the PLoS One. From my point of view, the authors should be complimented for their revisions made on the paper. Most of the reviewers’ comments were addressed in a satisfying manner. However, there are still some issues that are listed below and should be considered (lines refer to the revised manuscript with track changes):

**Response**

Thank you for your positive response and critical feedback that helps us to improve our paper. Lines refer to the version with track changes.

1) While I appreciate structuring the paper according to your two RQs, this is still missing in the abstract. Consider introducing RQ1 and RQ2 here as well. In doing so, the results within the abstract can me assigned to that structure easily. This would enhance understanding.

**Response**

References to RQ1 and RQ2 are now included within the abstract.

2) L.99: Although you almost entirely adjusted the naming of the criterion variable for RQ2 (competition performance), there are still some situation were you refer to level. Consider removing level here as well as in l.186 if appropriate. You are referring to a continuous variable in your analyses (performance score). Therefore, avoid using “level” which might suggest a nominal outcome. By the way: conder using “future competition performance” here.

**Response**

Thank you for your suggestions. We deleted 'level' at line 99, line 186 and line 298.

3) L.104 and throughout: check formatting of statistical values again. R^2 in italics?

**Response**

This has been changed as proposed.

4) L.187pp: while I appreciate your elaborations on the score, it would be helpful for the readership to provide a formula for this score, if possible. This would also help to get a better impression of your multilevel result…. If not just leave it as it is.

**Response**

Unfortunately, it is not possible to provide an easy calculation for the performance score. However, the complete description how the rating score is calculated can be found here: [De Elo-ranglijsten toelichting en berekeningswijze (nttb-ranglijsten.nl)](https://www.nttb-ranglijsten.nl/elo300.php). This webpage is in Dutch and provides a comprehensive outline. The link is added in the method section (line 190).

5) L.202 pp: Thank you for including information on KMC here. While this represents an interesting analysis, this- in the current version- does not refer to RQ1 or RQ2. Those RQs solely refer to the predictive value of the performed perceptuo motor test. Therefore I would suggest either including this analysis and their result into a RQ- presumably this could be RQ1a and the results referring to the tests could be RQ1b, or removing this analyses from the manuscript.

**Response**

We decided to delete the KMC in the revised version to focus directly on the proposed research questions.

6) L.210: provide more rationale why multi- and univariate approaches were followed a potential reason could be investigating single tests power (univariate), but also the test batterys power (multivariate). Just shortly elaborate on that, please.

**Response**

This information is added to the statistical analysis as proposed (line 211-214).

7) L.220: were these indeed different talent days for boys and girls? Or are the number 21 and 18 incidentally the same as those for the gender distribution? Please clarify. By the way: if there were different talent days, there is still further reason to discriminate boys and girls in your analyses: 1 year difference of testing dates, level of participants comparable among boys and girls?, …. Maybe at least add some thoughts to the discussion.

**Response**

The talent days included both boys and girls. Indeed the numbers are the same by coincidence. The exact numbers of boys and girls per year are added to the results section (line 223-224) to clarify this.

8) Table1: Please provide results for gender and age as covariates, please (as a follow-up in your answers to the latter revision). It is important to know, whether the covariates were significantly influencing the results, or not. Please state and also present as text in the results section. If there is a significant influence, you should also discuss whether there could be reasoned assumptions as to whether the prognostic relevance among genders could be different? [which would be maybe reflected by an interaction effect!?.

**Response**

Information about the covariates' significance in the univariable and multivariable models for both RQ1 and RQ2 is added in table 1 and 2 and in the text of the results section (line 243-245 and line 258-262). Based on these outcomes it appears that test age and sex were significant covariates within some of the univariable Cox regression analyses, but not in the final multivariable one. Influences of test age and sex are further discussed in the discussion section (line 300-305, line 327-332, line 337-341).

9) Table 1: Please provide at least 1 decimal for the Means of your test (in line with the given SDs that are already presented with 1 decimal.

**Response**

Decimals are now provided in Table 1 as proposed. These changes are not shown with track changes to preserve readability.

10) L.248: This subheading does not fit to the subheading of RQ1. Please use the same “structure”: should this also read “Predicting …” ?

**Response**

This has been changed as proposed.

11) L.250: delete “(p <.05”) as you present each exact p afterwards.

**Response**

This has been changed as proposed.

12) L.252: (Oxford) comma before “and eye-hand coordination!?

**Response**

This has been changed as proposed.

13) Table 2: Can you also present the R^2s for each model in the Table? This would be kind of an effect size and, thus, in line with all further analyses where you present effect sizes (e.g., HR).

**Response**

R^2^ have been added to table 2 as proposed.

14) L.317pp: As already mentioned, I appreciate presenting the limitations regarding sex and age. However, this is a severe issue in your analyses. Therefore, I recommend extending this section: what are your expectations about concrete limitations/restrictions of generalizability of your analyses towards the predictive value of the conducted tests. For me, it is by no means clear that the predictive power of such tests is the same for boys and girls. And by the way, this is well-known problem in talent research. Already William & Reilly (2000) claim for the consideration and comparison of both genders when investigating the predictive value of talent predictors. Just a thought: I know, that your sample size is too low for performing separate analyses (and getting significant results at the same time). However, did you perform those analyses also separately and compared e.g. the regression coefficients of those (non-significant) models with those given for the total sample. This (together with the results of the covariates) could indicate as to whether the differences between gender could be more or less neglected in your study. I would appreciate reading about such points here in this part of the discussion.

**Response**

As proposed, information about the covariates' significance in the univariable and multivariable models for both RQ1 and RQ2 is added in table 1 and 2 and in the text of the results section (line 243-245 and line 258-262). Based on these outcomes it appears that test age and sex were significant covariates within some of the univariable Cox regression analyses, but not in the final multivariable one. For the prediction of competition performance, only test age appeared a significant covariate. The issue of possible (interaction) effects of sex is pointed out in the discussion part (line 300-305, line 327-332, line 337-341).

Thank you again for this interesting paper. I hope that my comments are appreciated and help to further enhance the quality of the paper.

**Response**

Thank you for your feedback, this is much appreciated.
